# SIP SMART: a parallel group randomised feasibility trial of a tailored pre-treatment swallowing intervention package compared with usual care for patients with head and neck cancer

**DOI:** 10.1186/s12885-020-06877-3

**Published:** 2020-04-29

**Authors:** Roganie Govender, Christina H. Smith, Helen Barratt, Benjamin Gardner, Stuart A. Taylor

**Affiliations:** 1 Research Department of Behavioural Science & Health, University College London and Head & Neck Cancer Centre, University College London Hospital, 250 Euston Road, Ground floor Central, London, NW1 2PQ UK; 2 Division of Psychology & Language Sciences, University College London, London, UK; 3 NIHR CLAHRC North Thames, Department of Applied Health Research, University College London, London, UK; 4 Department of Psychology, Institute of Psychiatry, Psychology and Neuroscience (IoPPN), Kings College London, London, UK; 5 Centre for Medical Imaging, University College London, London, UK

**Keywords:** Head and neck cancer, Swallowing, Dysphagia, Behaviour change intervention, Feasibility study, Randomised controlled trial

## Abstract

**Background:**

Dysphagia or difficulty in swallowing affects quality of life for most patients with head and neck cancer. SIP SMART – [Swallowing Intervention Package: Self-Monitoring, Assessment, Rehabilitation Training] aims to improve post-treatment swallowing outcomes through a targeted and tailored pre-treatment intervention. This feasibility study assessed 1) recruitment and retention, 2) patient acceptability of randomisation and participation, 3) patient adherence, and 4) sought to identify a suitable primary outcome for a definitive trial, including sample size estimation.

**Methods:**

This two-arm parallel group non-blinded randomised feasibility trial took place within a head and neck centre at a teaching hospital in London, UK. Patients newly diagnosed with stage III/IV head and neck cancer were recruited and underwent 6-month follow-up. Patients were randomised to SIP-SMART or usual care via an online web-based system. SIP SMART comprised two 45-min consultations including a baseline clinical and instrumental swallowing assessment, relevant educational information, targeted swallowing exercises, and specific behaviour change strategies to increase exercise adherence. Usual care comprised a single session including a baseline clinical assessment and generic information about the likely impact of treatment on swallowing.

**Results:**

A total of 106 patients were identified at pre-screening, 70 were assessed for eligibility. Twenty-six patients did not meet eligibility criteria [0.37, 95% CI 0.27 to 0.49]. Five of 44 [0.11, 95% CI 0.05 to 0.24] eligible patients were not approached by researchers during clinic. Seven [0.18, 95% CI 0.08 to 0.33] of the 39 approached declined participation. Target recruitment (32 consented patients) was achieved within the timeframe. At 6-months 29/32 [0.91, 95% CI 0.76 to 0.97] patients remained in the trial. Acceptability of randomisation and participation in the intervention was favourable, and adherence to the exercises exceeded the pre-defined 35% minimum criterion. The MD Anderson Dysphagia Inventory swallow related quality of life measure was selected as the most suitable primary outcome for sample size estimation. No adverse effects arose from the intervention, or study participation.

**Conclusions:**

A definitive trial of the SIP SMART intervention compared to usual care is feasible and can be undertaken with patients with head and neck cancer treated within the NHS.

**Trial registration:**

ISRCTN40215425, registered retrospectively.

## Background

Difficulty in swallowing (dysphagia) affects approximately 60% of patients with head and neck cancer (HNC) at the time of diagnosis [1], and almost all patients who undergo treatment during their cancer care pathway [2]. Both the tumour itself, and treatments such as surgery and chemo-radiation therapy have a negative impact on eating, drinking and swallowing [3–5]. Swallowing is a highly coordinated muscular activity that is generally performed at a sub-conscious level by most people unless the mechanism is disrupted causing dysphagia. Swallowing may also be described as a sub-maximal process, in that the force required to swallow food is less than the capability of the swallowing muscles [6]. Given that HNC and its treatments are known to result in dysphagia, there may be scope for optimising swallowing physiology through prophylactic exercise interventions that increase the strength and range of movement of swallowing muscles. It seems plausible that exercises may increase physiological reserve and possibly delay or even avoid difficulties with swallowing both during and after cancer treatments. However, a Cochrane systematic review of prophylactic swallowing exercises reported uncertainty around their efficacy [7]. The Cochrane review called for new studies that addressed inconsistencies in the current literature, notably choice of swallowing exercise protocols, methods to address poor patient adherence, and variability in the type and timing of outcome measures, all of which undermine assessment of efficacy [7].

We have developed a new pre-treatment intervention (SIP SMART: Swallowing Intervention Package- Self Monitoring, Assessment, Rehabilitation Training), designed to address some of the shortcomings of previous clinical trials. The intervention development was informed by a series of studies including: a systematic review to identify behavioural strategies that could potentially promote patient adherence to swallowing exercises [8]; an in-depth interview study to establish patient reported barriers and facilitators to exercise adherence [9]; and a think-aloud study to explore the potential use of video-animation to improve *how* information about swallowing and dysphagia is conveyed to patients [10]. Further detail about the systematic development of SIP SMART using the Medical Research Council framework for the development and evaluation of complex interventions [11] and the Behaviour Change Wheel [12] is described elsewhere [intervention development manuscript under review]. This new intervention has been designed to fit into the typical pathway for a patient being treated for HNC in the English National Health Service (NHS). Proof of efficacy would require randomised trial evidence compared to standard care. To our knowledge, there has been no previous randomised trial of a pre-treatment behavioural swallowing intervention for patients with HNC in the UK, and given the relative complexity of the intervention, and target patient group, feasibility testing was deemed essential. For example, it is unknown whether it would be feasible to recruit and randomise newly diagnosed patients, and whether the SIP SMART protocol could be accommodated within the short timeframe between diagnosis and the start of cancer treatment. In support of testing feasibility, a recent UK study on tube feeding in HNC patients highlighted the difficulties in recruiting and randomising patients before treatment [13]. The present study sought to assess the feasibility of a future definitive trial, specifically testing recruitment and retention, patient acceptability of randomisation and participation, patient adherence, and to identify a suitable primary outcome and inform sample size estimation.

## Methods

A full protocol of this study has been published [14]. In summary, this was a two-arm parallel group non-blinded randomised controlled feasibility trial allocated in a 1:1 ratio. Patients were stratified by first line treatment (surgery or radiotherapy with or without chemotherapy), and random block permutations were used to ensure a balance between both trial arms. The study took place at a single NHS hospital site within a dedicated HNC service. The study received full ethical approval from the NRES committee London (14/LO/0175) and was registered on the trials Database (ISRCTN40215425).

### Study sample and inclusion/exclusion criteria

Sample size was determined pragmatically using guidance that *n* of 30 is usually sufficient to estimate key parameters in a feasibility study [15]. Local audit data suggested that approximately 70 patients with newly diagnosed stage III and stage IV HNC were referred to the recruitment site each year. We therefore estimated that it would take approximately eight months to enrol 32 patients in this study assuming a minimum of 60% of eligible patients were recruited.

Patients were eligible for inclusion if they were: 1) newly diagnosed with Stage III or IV HNC; 2) discussed at the head and neck multidisciplinary meeting and referred for treatment via surgery and/or chemo-radiotherapy or combinations thereof; 3) aged 18 and above and able to provide informed consent; and 4) sufficiently proficient in English to participate and engage in the intervention. Patients were excluded if they: 1) had had previous treatment for head and neck cancer; 2) were either mid treatment or being treated with palliative intent; 3) underwent solely non-standard treatment such as brachy therapy, photodynamic therapy or chemotherapy alone; 4) were planned for total laryngectomy; 5) were considered vulnerable, had significant co-morbidities or were unable to provide informed consent; or 6) had brain tumours and other primary sites not within the head and neck.

### Patient identification and consent

Any member of the multidisciplinary team (MDT) initially identified potential patients from the case discussions at the weekly head and neck meetings. The treating consultant introduced the SIP SMART trial to prospective patients during their clinic consultation. The researcher (RG) or research nurse subsequently approached the patient to discuss the trial in more detail. Patients were provided with the patient information leaflet and advised that they would be contacted over the next 2 days to answer any further questions and/or to discuss participation and consent. As patients were usually required to attend the hospital for other tests, either the researcher or research nurse could arrange a further face-to-face meeting to obtain informed consent, if this was appropriate. Randomisation took place immediately after written consent was obtained. An online computer generated service with immediate allocation was used (http://www.sealedenvelope.com/). This procedure ensured that allocation was revealed to the researcher and patient at the same time. Patients who were allocated to the intervention group were advised that they would be booked for an x-ray swallow investigation as part of the new intervention, while those allocated to usual care were informed that a speech and language therapist would contact them for an appointment within a few days.

### Interventions and procedures

#### Usual care group

Local specialist head and neck speech and language therapists (SLTs) provided usual care based on a usual care manual devised and agreed by the therapists prior to the commencement of the trial. The usual care pre-treatment consultation consisted of one 45-min session which included: general history taking and introducing the patient to the SLT role; clinical baseline screening of swallowing and communication using pre-specified measures such as the Water Swallow Test (WST), the Performance Status Scale (PSS) and the MD Anderson Dysphagia Inventory (MDADI); and information provision about the general impact of treatment on swallowing function and the likely side effects and anticipated changes to swallowing and communication if relevant. Patients planned for chemo-radiation treatment were given general advice on dealing with dry mouth and taste changes, and a generic handout of prophylactic swallowing exercises such as the effortful swallow and the Shaker exercise (head-lift exercise) that they were advised might be helpful.

#### Intervention group

This group received the SIP SMART intervention, described more comprehensively elsewhere [manuscript under review]. In summary, the intervention took place over two 45-min consultations that followed each other on the same day or with a day between them depending on patient preference. As part of SIP SMART, patients underwent an x-ray swallow assessment that enabled a physiological analysis of swallowing and the selection of specific and targeted exercises. Patients were shown a video-animation of swallowing to anchor the pre-treatment discussion, making it more concrete for the patient and promoting an interactive approach [10]. Specific behaviour change techniques such as goal setting, self-monitoring, and behavioural practice were actively employed. These were postulated to increase patient engagement and possibly adherence with prophylactic swallowing interventions [8].

Following cancer treatment, patients in both groups received the usual post-treatment swallowing rehabilitation offered at the centre. All patients were followed up for a 6 month period, with outcomes collected during follow-up visits at one, three and 6 months after the completion of cancer treatment. The complete list of measures is included as supplementary information (Additional file 1; see also reference [14]).

### Feasibility outcomes

The main criteria to determine the feasibility of a definitive trial were a priori defined as:

1. *Recruitment:* Achieving a target recruitment of 32 patients over an eight-month period. Total accrual, and rate of accrual were measured using screening and enrolment forms.
2. *Acceptability:* Generally positive patient perceptions of randomisation between interventions, and toward participation – This was determined via a published questionnaire using simple frequency counts for patient responses (yes, no, unsure) [16].
3. A minimum of 35% of the intervention group reporting satisfactory to good adherence to the requirements of the intervention – A study questionnaire was devised to test this. The 35% threshold was chosen based on the highest adherence reported in the literature at the time of trial design by studies undertaking a similar swallowing exercise intervention over a protracted time-frame [16]. The adherence form was given to both groups, as patients in the usual care group also received a sheet of generic exercises at their pre-treatment consultation. Adherence data was gathered at 1-month, 3-months, and 6-months and reflected a composite estimate based on patient reports of their exercise adherence over the preceding month.
4. Identification of a suitable outcome measure and sample size calculation – A number of potential primary outcomes were tested so the most appropriate could be identified based on achieving high data completeness (> 70%) [17], while measuring a clinically important swallowing outcome (preferably with a known minimum clinically important difference or MCID), and leading to a pragmatically achievable sample size estimate.

### Analysis

The Consort flowchart was used to report recruitment and retention figures. Descriptive statistics including means, standard deviations and confidence intervals were calculated for all potential outcome measures. Cohen’s d was used to estimate effect sizes or potential magnitude of any differences in swallow related outcomes between SIP SMART and usual care. Sample size was calculated based on mean estimates for a parallel group RCT with a continuous outcome, 95% confidence interval (alpha level = 0.05) and power of 80%.

Qualitative information was collected through a researcher diary used to record observations about the research process and data collection that might help explain findings and/or provide useful information for a future trial.

## Results

Preliminary results of this study have been previously reported and published as an oral presentation conference abstract [18]

### Screening and recruitment

**Fig. 1:**
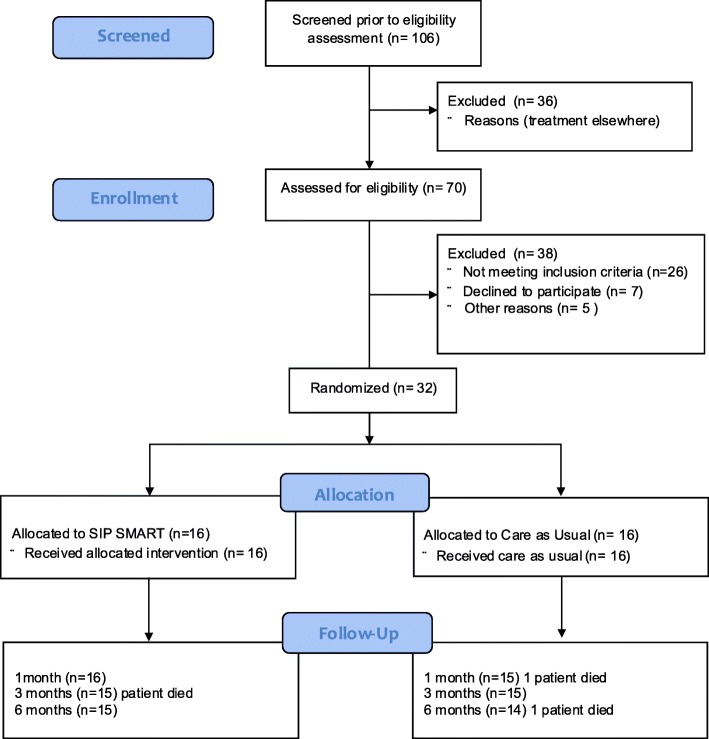
Consort diagram illustrating flow of patients through the SIP SMART trial

A total of 106 patients were identified as potentially eligible from the weekly MDT list, based on the pre-screening (i.e. eligible diagnosis) (Fig. 1). However, 36 (34%) were excluded after MDT discussion. Service re-configuration changes implemented after the study was planned meant that these patients were offered and received treatment at other hospital sites closer to their home. As this feasibility trial had approval for a single site only, these patients were not approached for participation. Of the remaining 70 patients who met the criteria for pre-screening based on diagnosis, a total of 26 [0.37, 95% CI 0.27 to 0.49] patients were subsequently excluded following eligibility assessment; eleven [0.42, 95% CI 0.26 to 0.61] patients had unsatisfactory proficiency in English determined by the requirement for an interpreter at consultations. Six [0.23, 95% CI 0.11 to 0.42] patients presented with medical co-morbidities and pre-existing dysphagia unrelated to HNC. Nine [0.35, 95% CI 0.19 to 0.54] patients had to have a change in treatment (for example, total laryngectomy, or treatment with palliative intent). Of the remaining 44 eligible patients, five [0.11, 95% CI 0.05 to 0.24] were not approached in the clinic as they left prior to be seen by the researcher. Seven [0.18, 95% CI 0.08 to 0.33] of the 39 patients approached declined participation. There was some overlap in the reasons provided, but the main stated reasons were: not wanting to be involved in anything extra while having treatment (3/7) [0.43, 95% CI 0.16 to 0.75]; concern about additional hospital visits (2/7), [0.29,95% CI 0.08 to 0.64] and pre-existing participation in another trial (2/7) [0.29,95% CI 0.08 to 0.64]. None of the patients reported declining based on anything in the patient information leaflet or any concerns about the intervention itself. Thus, of the 39 eligible patients approached during the “face to face” clinic visit, 32 [0.82, 95% CI 0.67 to 0.91] were recruited.

**Fig. 2:**
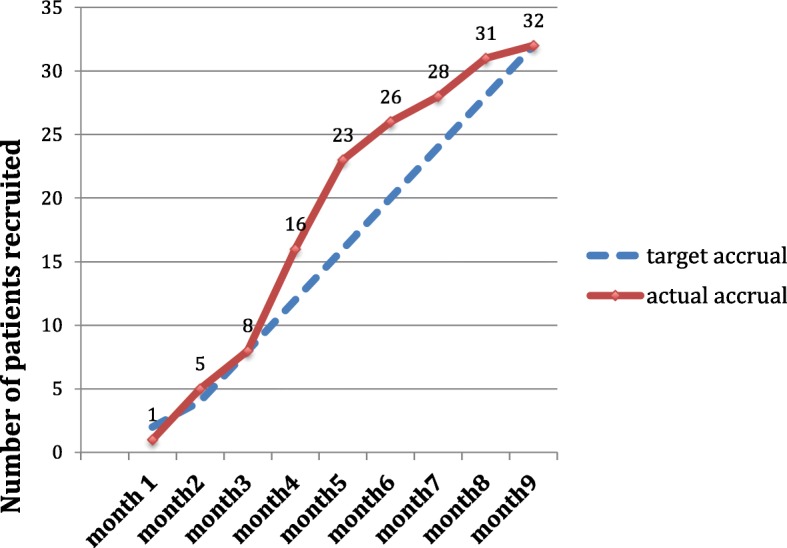
Accrual into the SIP SMART feasibility trial

Figure 2 illustrates accrual to the SIP SMART trial over an 8-month duration from mid April –mid December (spanning 9 calendar months). The pattern shows a steady rate of recruitment following an initial slower start. The target recruitment (based on prior knowledge of referral patterns into the centre) of an average of four patients a month was shown to be feasible.

### Patient characteristics

The baseline characteristics of all patients recruited to the study are displayed in Table 1. Despite the small sample size, the groups were generally well balanced, and any differences likely due to chance. The average age of participants across the groups was 57 years with five times as many males than females recruited, consistent with the general disease prevalence. The sample included the most common curative options for treatment, with stratification by first line treatment ensuring that a balance between the groups was achieved. Baseline weight and body mass index were comparable across groups. Information regarding baseline smoking and alcohol history has not been included in the summary table as this information was inconsistently reported on the case report forms.

**Table 1:** Patient characteristics

Participant demographics	Intervention group (***n*** = 16)	Care as usual group (***n*** = 16)
Age mean (SD)	58.56 (12.41)	55.19 (9.45)
Gender *n* (%)		
Male	15 (94%)	12 (75%)
Female	1 (6%)	4 (25%)
Ethnicity *n* (%)		
White	11 (69%)	12 (75%)
Asian/Asian British	3 (19%)	1 (6%)
Black/ Black British	0 (0%)	1 (6%)
Chinese	2 (12%)	0 (0%)
Other	0 (0%)	2 (13%)
Marital status *n* (%)		
Married	4 (25%)	10 (63%)
Single/separated	5 (31%)	5 (31%)
Widowed	1 (6%)	0 (0%)
Co-habiting	2 (13%)	1 (6.%)
Divorced	4 (25%)	0 (0%)
Employment status n (%)		
Full-time	7 (44%)	1 (6%)
Part-time	3 (19%)	1 (6%)
Self-employed	1 (6%)	5 (31%)
Not employed	3 (19%)	6 (38%)
Retired	2 (12%)	3 (19%)
Occupation *n* (%)		
Manager/director	2 (13%)	1 (6%)
Graduate professional	4 (25%)	0 (0%)
Associate professional/technical	1 (6%)	0 (0%)
Admin/secretarial	1 (6%)	2 (13%)
Skilled trade	2 (13%)	5 (31%)
Sales/customer services	1 (6%)	1 (6%)
Caring/leisure	4 (25%)	6 (38%)
Other	1 (6%)	1 (6%)
AJCC numeric tumour stage		
III	6 (37.5%)	6 (37.5%)
IV	10 (62.5%)	10 (62.5%)
Tumour site *n* (%)		
Oral cavity	5 (31%)	3 (19%)
Nasopharynx	1 (6%)	0 (0%)
Oropharynx	9 (56%)	10 (62%)
Hypopharynx/larynx	1 (6%)	3 (19%)
Cancer treatment *n* (%)		
Surgery	1 (6%)	0 (0%)
Radiotherapy	4 (25%)	1 (6.%)
Surgery & radiotherapy	1 (6%)	2 (12%)
Radiotherapy & chemotherapy	9 (56%)	11 (69%)
All three	1 (6%)	2 (13%)
Other treatment *n* (%)		
Nasogastric tube	4 (25%)	4 (25%)
Gastrostomy tube	10 (62%)	11 (69%)
Neither/NA	2 (13%)	1 (6%)
Weight Baseline mean (SD)	72.46 (15.37)	78.46 (15.36)
BMI Baseline mean (SD)	24.71 (3.77)	27.14 (4.19)

### Patient acceptability of participation and randomisation

Table 2 shows responses from 24 patients who returned the questionnaire, categorised into agree, unsure, disagree with the given statements. Patients reported high agreement with several statements indicating they felt that the trial offered the best treatment (SLT intervention) available (100%), they were satisfied that either treatment group would be suitable (83%), were given sufficient information about the trial (88%) and were aware that they could leave the trial at any time without their care being compromised (100%). Given a choice, just over half of patients indicated that they wanted their doctor or health care professional to choose their treatment rather than being randomised by a computer. Four patients (17%) reported being worried by the idea of randomisation. One patient reported feeling unable to decline participation but was aware that it was possible to leave the trial at any stage. All patients indicated that they wanted to help with the research, and felt that other patients would benefit from the results.

**Table 2:** Patients’ responses to trial participation questionnaire

No	Statement	Agree			Unsure			Disagree		
		%	CAU	INT	%	CAU	INT	%	CAU	INT
1	I thought the trial/study offered the best treatment available	**100**	12	12	**–**	–	–	**–**	–	–
2	I believed the benefits of treatment in the trial would outweigh the side effects.	**71**	6	11	**29**	6	1	**–**	–	–
3	I was satisfied that either treatment in the trial would be suitable.	**83**	10	10	**13**	1	2	**4**	1	–
4	I was worried that my illness would get worse unless I joined the trial.	**17**	2	2	**8**	2	–	**75**	8	10
5	The idea of randomization worried me.	**17**	4	–	**13**	1	2	**70**	7	10
6	I wanted a doctor to choose my treatment rather than randomized by computer	**54**	8	5	**21**	4	1	**25**	0	6
7	The doctor told me what I needed to know about the trial.	**67**	9	7	**13**	2	1	**20**	1	4
8	I trusted the doctor treating me.	**96**	12	11	**–**	–	–	**4**	–	1
9	I was given too much information to read about the trial.	**42**	7	3	**13**	–	3	**45**	5	6
10	I was given enough information to read about the trial.	**88**	11	10	**8**	1	1	**4**	–	1
11	I knew I could leave the trial at any time and still be treated.	**100**	12	12	**–**	–	–	**–**	–	–
12	I did not feel able to say no.	**4**	1	–	**8**	2	–	**88**	9	12
13	I wanted to help with the doctor’s research	**100**	12	12	**–**	–	–	**–**	–	–
14	I feel that others with my illness will benefit from the results of the trial.	**100**	12	12	**–**	–	–	**–**	–	–
15	The doctor wanted me to join the trial.	**38**	4	5	**37**	5	4	**25**	3	3
16	Others, for example, family or friends, wanted me to join the trial.	**42**	7	3	**12**	3	–	**46**	2	9

Note: % = overall percentage from all respondents, *CAU* number in care as usual group, *INT* number in intervention group

### Adherence to intervention

**Fig. 3:**
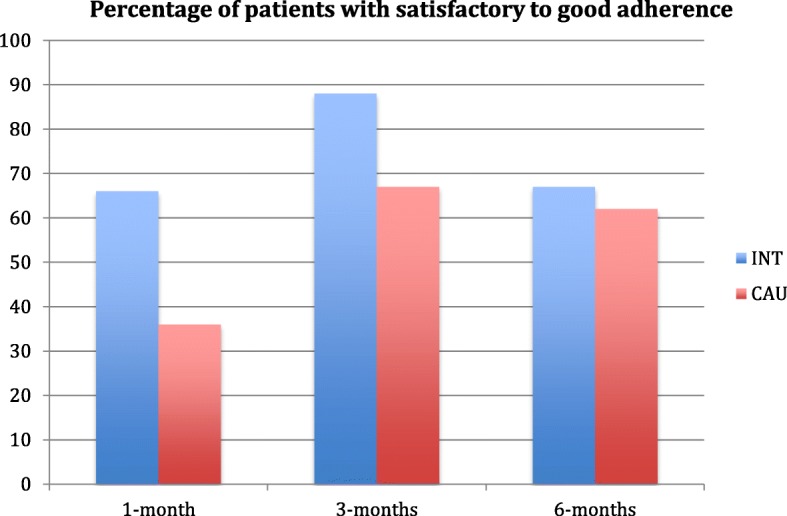
Patient reported adherence (INT = intervention, CAU = usual care)

The completeness of adherence data was 77% (24) at 1 month, 70% (21) at 3 months, and 83% (24) at 6 months, after accounting for the three deaths. Figure 3 shows the percentage of patients within each group who demonstrated satisfactory to good adherence based on the responses to the adherence form.

Satisfactory to good adherence was greater than the 35% minimum threshold for the intervention group across all time-points thus meeting the stipulated criterion. The results also show reports of good adherence from the usual care group, who were given a generic exercise sheet.

### Candidate outcome measures, data completeness and sample size estimation

Table 3 shows that the minimum threshold of 70% was achieved for most measures within both study groups except for the water swallow test (WST). Three patients declined to have the 6-month MBS; two reported that their swallowing had improved and they could not attend the appointment within the required timeframe and the other reported that he did not wish to have further exposure to radiation after completing his treatment.

**Table 3:** Completeness of swallowing outcome measures across time-points for both groups

Measure	Obtained Intervention [INT] no (%)	Obtained Care as usual [CAU] no(%)	Comments or reasons for non-completion – (researcher diary)
Baseline (T0) [expected INT = 16, CAU =16]			One patient did not complete questionnaires at appointment, and did not respond to requests to return them at next visit.
FACT	16 (100)	15 (94)	
MDADI	16 (100)	15 (94)	
PSS (normalcy of diet)	16 (100)	16 (100)	
100 mL WST	16 (100)	16 (100)	
FIGS (swallowing)	16 (100)	16 (100)	
MIO (jaw opening)	16 (100)	16 (100)	
One Month (T1) [expected INT =16, CAU = 15]			*100 mL WST - Clinical notes indicate that several patients reported that they were unable to drink continuously at 1-month post treatment, or clinicians felt it was unsafe to ask them to do so. These patients were given a score of zero and still rated complete.
FACT	13 (81)	15 (100)	
MDADI	13 (81)	15 (100)	
PSS	14 (88)	14 (93)	
100 mL WST	*13 (81)	*10 (67)	
FIGS	14 (88)	14 (93)	
MIO	14 (88)	12 (80)	
Three Months (T2) [expected INT = 15, CAU = 15]			*100 mL WST – As above. Additionally, it was noted that clinicians sometimes did not attempt this measure if the water cooler in the clinic was not working (*n* = 3) – availability of resources.
FACT	11 (73)	12 (75)	Four patients did not attend their 3-month follow-up visit, but did not wish to drop out of the study.
MDADI	11 (73)	12 (75)	
PSS	11 (73)	8 (53)	
100 mL WST	*8 (53)	*8 (53)	
FIGS	11 (73)	8 (53)	
MIO	11 (73)	8 (53)	
Six Months (T3) [expected INT =15, CAU =14]			Improvement in collection of most measures in comparison to 3-months as patients were attending for their MBS swallow assessment at the same time as clinical measures were taken.
FACT	12 (80)	13 (93)	Due to an unavoidable technical issue (power surge causing loss of exams) **MBS exams could not be rated for 7 patients (INT =4, CAU =3), although for both groups > 70% completed the procedure.
MDADI	12 (80)	13 (93)	
PSS	13 (87)	12 (86)	
100 mL WST	13 (87)	12 (86)	3 patients declined to have the 6-month MBS (INT = 2, CAU =1)
FIGS	13 (87)	12 (86)	
MIO	13 (87)	12 (86)	
**MBS Impairment Profile Score	9 (60) 87	10 (71) 93	
PAS Score	9 (60) 87	10 (71) 93	

Notes: *FACT* Functional Assessment of Cancer Therapy, *MDADI* MD Anderson Dysphagia Inventory, *PSS* Performance Status Scale, *WST* Water Swallow Test, *FIGS* Functional Intra-oral Glasgow Scale, *MIO* Maximum Incisor Opening, *MBS* Modified Barium Swallow, *PAS* Penetration-Aspiration Scale

For the remaining outcome measures, complete datasets across the four time-points were available for eight patients in the intervention group and 11 in CAU. Excluding the three patients who died, this figure represents an overall total of 66%, [95% CI 0.47–0.80]. If only the baseline and the 6-month endpoint are considered, the figure increases to 86%, [95% CI 0.70–.0.94]. This means that despite having multiple outcome measures, data completeness was at 86% when considering the baseline and the 6-month endpoint. While none of the patients dropped out of the study, loss of data due to missing information was 14% [95% CI 0.06–0.30] when considering the baseline and 6-month endpoint.

Table 4 shows that effect sizes [19] were large (≥ 0.8) for the Functional Assessment of Cancer Therapy (FACT-HN) and moderate (≥ 0.5) for the MD Anderson Dysphagia Inventory (MDADI), Maximal Incisor Opening (MIO) and Functional Intra-oral Glasgow Scale (FIGS).

**Table 4:** Main swallow-related outcome measures and effect sizes (mean, standard deviation and 95% confidence intervals at baseline and final time-point included)

Outcome measure	Group	Baseline (T0) mean (±95% CI) ***SD***	Between-group effect size (Cohen’s d)	6-months (T3) mean (±95% CI) ***SD***	Between-group effect size (Cohen’s d)
**FACT-H&N and MDADI: [n (baseline) intervention = 16 CAU = 15; n (T3) intervention = 12 CAU = 13]**					
FACT-H&N total score	Intervention	104.38 (91.74–117.02) *23.71*	0.14	89.98 (79.24–100.72) *16.9*	0.83
	Care as usual	101.28 (89.03–113.54) *22.13*		76.9 (68.02–85.78) *14.69*	
MDADI Composite	Intervention	83.49 (75.17–91.81) *15.62*	0.36	69.74 (56.42–83.05) *20.95*	0.61
	Care as usual	77.82 (68.93–86.72) *16.06*		59.35 (52.03–66.67) *12.11*	
**Other measures: [n (baseline) intervention = 16 CAU = 16; n (T3) intervention = 13 CAU = 12]**					
MIO - Jaw opening	Intervention	46.25 (39.44–53.06) *12.78*	−0.15	43.00 (37.24–48.76) *9.53*	0.68
	Care as usual	47.81 (43.83–51.99) *9.84*		34.33 (24.56–44.1) *15.38*	
PSS HN Normalcy of diet	Intervention	70.00 (56.24–83.76) *25.82*	−0.22	70.00 (52.21–87.79) *29.44*	0.30
	Care as usual	75.63 (62.43–88.82) *24.76*		60.83 (41.21–80.46) *30.88*	
FIGS Swallowing	Intervention	4.25 (3.84–4.66) .*78*	−0.35	4.15 (3.42–4.89) *1.21*	0.53
	Care as usual	4.50 (4.11–4.89) *.73*		3.50 (2.71–4.29) *1.24*	
**MBS and PAS at T3: (intervention = 9 CAU = 10) – not performed for both groups at baseline**					
PAS	Intervention			3.67 (1.24–6.1) *3.16*	*0.13*
	Care as usual			3.3 (1.36–5.24) *2.71*	
MBS Imp (composite)	Intervention			6.44 (4.49–8.38) *2.53*	*0.24*
	Care as usual			5.96 (5.05–6.86) *1.27*	

### Sample size calculation

Due to the low numbers available for analysis on the MBS composite score at 6-months, it was not possible to reliably estimate sample size for this measure. The effect size of the PSS was found to be small, meaning that sample size of 340 (+ 82, accounting for 24% attrition) would be required to detect clinically important changes. The MDADI met most criteria for a primary outcome measure and showed a moderate effect size. On balance from the measures available, the MDADI was considered the most suitable choice for a primary outcome (see Discussion). Sample size was therefore estimated for the MDADI patient reported outcome. Using the data obtained from the feasibility study, the sample size required if the MDADI were the primary outcome would be 86 (43 in each group). Based on this feasibility study, this figure will need to be inflated by 10% for attrition due to death and an estimated 14% due to missing data. A sample size of 106 will therefore be required for a future 2-arm parallel group trial.

### Harms

There were no unexpected serious adverse events related to the trial.

## Discussion

SIP SMART is a pre-treatment swallowing intervention for newly diagnosed head and neck cancer patients. All four main criteria specified in the protocol to determine success from the feasibility trial were satisfied: the target recruitment (32 patients) was achieved at an average rate of four patients a month; the MDADI was identified as a suitable primary outcome for which sample size was estimated; patient responses to the questionnaire on acceptability and randomisation was mainly positive; and the minimum reported adherence for the intervention group was attained.

The recruitment strategy of using the weekly multidisciplinary meeting list for pre-screening worked well and would be relatively straightforward to implement in a larger multi-centre trial. Mapping the different treatment pathways that exist at each centre, and clarifying how these will impact eligibility for inclusion in the trial will help to avoid over-estimating recruitment potential. The researcher and research nurse found it useful to identify potential patients to the treating consultant at the start of the clinic, and to ensure a study card was attached to the front of the medical notes so that the researcher could be alerted when the patient was being seen. Whilst this worked for the current study, we recognise that many hospitals are moving toward electronic health record systems, which may make it easier for potential participants to be identified. Given the narrow window of opportunity to enrol patients into this pre-treatment intervention study, these strategies should be fully explored for each site involved in a future trial ensuring full MDT support to achieve good recruitment. Patients without adequate proficiency in English were excluded from the current study given the nature of the intervention. However, a sizable number of the patients who were not eligible (11/26, 42%) were excluded due to language barriers, making it important to consider whether a future trial should cost for interpreter services.

Participant characteristics were broadly reflective of the population of patients who present with HNC. There was a higher incidence of males compared to females, although the male:female ratio across oral and oropharyngeal cancers is approximately 3:1 [20–22]. The average age (57 years) reflects the typical patient demographic. The sample included a higher proportion of patients with oropharyngeal cancer that tends to be associated with the human papillomavirus and often manifests before 60 years of age [20]. It is possible that this demographic may differ in parts of the country where other etiological factors (for example, chewing betel products) play a more dominant role in cancer incidence [23]. In our sample, there was a higher proportion of patients who received chemo-radiation therapy compared with surgery as first line treatment as this is the standard care for the tumour site (mainly oropharyngeal) and tumour stage (Stage III/IV) mostly found in our sample [24]. The placement of a prophylactic feeding tube for individuals with advanced tumours undergoing chemo-radiation is in keeping with the practice guidelines at the institution.

Choosing the type and timing of primary outcome measure was an important outcome for this feasibility trial. Patients treated for HNC usually show a decline in swallowing function in the immediate post-treatment phase with gradual recovery over the following months until more stable functioning is achieved at around 6-months after treatment [25, 26]. The pattern of data for all swallowing outcome measures in this feasibility study showed a similar trend. It may therefore be appropriate to consider whether the research process for the future trial would be better served by having just two time-points (baseline and 6-month) for data collection, thereby ensuring a higher percentage of data completeness as recorded in this trial. Guidelines suggest that a 70% completion rate for studies with 6-month or longer follow-up would be satisfactory [17].

A known minimum clinically important difference (MCID) is helpful in estimating sample size for a primary outcome. Published data suggest that an actual difference of 5–12 points on the FACT-HN [27] and 10 points on the MDADI and PSS-HN (Performance Status Scale) are generally regarded as clinically meaningful [28]. The FACT-HN demonstrated the best effect size but may be too generic and more suitable as a secondary outcome. MIO focuses on jaw opening only and is therefore too narrow to capture swallowing function but could provide useful information as a secondary measure. MIO is perhaps the easiest measure by which to observe changes that may occur if patients adhere well to their exercises. While the MBS composite score might be the most appropriate measure of swallow physiology, the scoring system is yet to be validated, and it was much more challenging to obtain complete data on this measure. The decision to select the MDADI as the outcome measure for sample size estimation was therefore informed by several factors: it is a validated patient reported measure, has a known MCID, shown to have excellent completion rates in the feasibility study and has an achievable sample size for a definitive trial of SIP SMART.

The majority of patients reported positive views toward participation in the trial and were not worried by the idea of randomization. The method used for randomisation was well received by both patients and clinicians, as it was transparent and done immediately after consent. Previous research has indicated that patients may decline participation in trials if they have a preference for one arm of the trial or if they are worried about the idea of randomisation [16]. Based on questionnaire responses, this was not a major concern for SIP SMART. Patients seemed to have understood the concept of chance and were satisfied that either the new intervention or usual care would be suitable. In general, the reasons reported for participation in this trial were consistent with those reported in other cancer trials [16]. On the whole, patients newly diagnosed with HNC are willing to participate in this type of trial often for altruistic reasons. It is therefore expected that patients would be no less likely to agree to participate in a future larger trial, despite recruitment occurring at a difficult time pre-treatment.

This feasibility study made use of a study specific form to capture information on exercise adherence. The target was to obtain a minimum of 35% of patients achieving at least satisfactory to good adherence to the intervention based on figures reported in a previous similar trial [16]. There remains much debate about how to measure adherence to swallowing exercises. Some researchers have suggested that adherence should be measured in terms of both frequency and intensity of exercises, highlighting the challenges for measuring adherence in clinical trials [29, 30]. It is possible that the measurement method used in this study was too generalized, and that adherence was overestimated. In the absence of any formal measure, the study specific form devised for SIP SMART used a combination of questions (reflecting frequency and intensity) to classify responses into satisfactory to good versus poor to no adherence. Further work may be necessary to ascertain whether a more suitable method may be available to capture adherence in a larger trial or whether the current method provides a good enough reflection of adherence given that most current methods rely on patient self-report. Researcher effort and patient burden involved in obtaining more detailed and specific adherence information will be important considerations when making this decision in a future trial.

While no direct safety concerns or harms arose from the feasibility trial, it is important to recognize that patients participating in such a behavioural intervention may feel under immense pressure if they are unable to adhere to their swallowing exercises. Feelings of guilt or hopelessness may surface, and it is imperative that mechanisms are in place to identify and counteract these feelings. A problem solving, facilitative approach was built into the intervention design to mitigate such feelings, and regular appointments with the team acted as an important safeguard. The modified barium swallow procedure was identified in the protocol as a possible source of harm if contrast material was inhaled. However, given that the modified barium swallow protocol starts with very small amounts of contrast (5 mL) compared with that given during a standard barium swallow, patients at risk for airway compromise can usually be identified before they are asked to swallow any substantial volumes.

Study limitations must be acknowledged**.** This study was conducted as part of a fellowship in which the intervention developer (RG) was also involved in recruitment and was therefore a key driver in the study. As an invested recruiter, this may have increased success with recruitment. For this success to be replicated, it will be crucial to identify key individuals (principal investigators) to drive the project and enlist a similar level of multidisciplinary support at other sites. Failure to ensure this could be met with poorer recruitment in a larger trial.

Our inclusion/exclusion criteria was as per protocol, and we therefore cannot be sure of how the results may differ if the excluded groups are included. Furthermore, our sample had small numbers for some tumour sub-sites such as nasopharynx. Given that this was a feasibility trial, a larger trial would be necessary to consider differences in different cancer sub-groups.

For the feasibility trial, implementation of the intervention was done solely by the clinical researcher and was therefore well controlled. The researcher was able to accommodate performing the pre-treatment MBS that was part of the new intervention at short notice. Patients in the usual care group did not receive a baseline MBS, as it was not part of usual care. This would have required additional resources from the clinical team. Practical implementation of the protocol will therefore need to be carefully planned for each site taking into account available resources. Further to this, it will be necessary for some training in the new intervention so that it can be delivered with good fidelity. These aspects have not been addressed by the current feasibility study.

Patients, clinicians and the researcher were all aware of group allocation as blinding was not attempted in this feasibility trial. The researcher was not involved with the collection of outcome data (questionnaires and clinical measures were collected by the research nurse/other SLTs) and made every effort to maintain distance until the completion of the study. Improved processes to ensure blinding should be further explored prior to a definitive trial.

Practical constraints imposed limitations on the study design and procedures. For example, conducting an in-depth exploration of patient experiences after participation could have provided useful insights for further refinements of the intervention itself and/or the study processes. However, given the time constraints this could not be accommodated as part of the current programme of work. It may be possible to do this as part of further pilot work.

Three patients died (approximately 10%) over the duration of this trial, an important consideration given the nature of the disease and expected survival. Whilst none of these deaths were related to the SIP SMART intervention, attrition due to death is an important consideration for the future trial. To our knowledge, all patients who provided final outcome data at the end of the study were tumour-free, based on their last multidisciplinary team review. This study did not distinguish between cancers that were HPV positive (better prognosis and survival) and those that were not. A study sample with greater numbers of advanced cancer due to non-HPV positive disease may therefore show greater attrition due to generally poorer survival.

Several study-related forms were devised for the feasibility study and most could be used in a larger trial. Some may require modification. The case report form, for example, was detailed, and it may be possible to reduce the amount of information collected. In this study, information on smoking and alcohol could be reduced to simpler yes/no questions without the level of detail included in the current case report form.

Many previous studies of swallowing exercise interventions in the head and neck population have been reported as definitive trials drawing conclusions about efficacy despite frequent inadequate sample sizes [31]. The focus of our study has been on feasibility outcomes that will optimise the planning and conduct of a future trial, and therefore does not report on intervention efficacy at this stage. It is encouraging to note that since our initial work on this topic, a more recent meta-analysis of swallowing exercise interventions in the head and neck population has demonstrated that exercises both before and after chemoradiation treatment show improvements in jaw opening and swallow function, with early interventions also having a positive effect on airway protection during swallowing [31]. Although the review authors found no evidence for improved quality of life outcomes for either early or late interventions, their meta-analysis and recommendations render further support for more well-designed trials on this subject.

## Conclusion

SIP SMART is a swallowing pre-habilitation intervention that can be accommodated in a typical NHS head and neck cancer pathway, and is feasible for the conduct of a definitive trial to examine its efficacy relative to current practice. A few important issues need to be addressed prior to proceeding to a main trial. This includes training of speech and language therapists to deliver the intervention and addressing any concerns around practical implementation of the protocol at different hospital sites. Such issues could be addressed in a small-scale multi-centre pilot study prior to progression to a definitive trial.

## Supplementary information

**Additional file 1.** Outcome measures and timepoints for SIP SMART trial.

## Data Availability

All data generated or analyzed during this study are included in this published article and its supplementary information files.

## Abbreviations

CAU: Care as usual/usual care
FACT-HN: Functional assessment of cancer therapy, head and neck
HNC: Head and neck cancer
HPV: Human papilloma virus
MBS: Modified barium swallow
MBS ImP: Modfied Barium Swallow Impairment Profile
MCID: Minimum clinically important difference
MDADI: MD Anderson Dysphagia Inventory
MDT: Multidisciplinary team
MIO: Maximal incisor opening
NHS: National Health Service
PAS: Penetration aspiration scale
PSS-HN: Performance Status Scale for head and neck
SIP SMART: Swallowing Intervention Package: self-monitoring, assessment, rehabilitation training
SLT: Speech and language therapy/therapist
WST: Water swallow test
