# Supplementary material for: SIP SMART: a parallel group randomised feasibility trial of a tailored pre-treatment swallowing intervention package compared with usual care for patients with head and neck cancer
Source: BMC Cancer. 2020 Apr 29;20:360. doi: 10.1186/s12885-020-06877-3 (PMC7191731; doi:10.1186/s12885-020-06877-3)
Supplement: Supplementary file 1 — Additional file 1. Outcome measures and timepoints for SIP SMART trial. [file 12885_2020_6877_MOESM1_ESM.docx]

**Additional File 1. Outcome measures and timepoints for SIP SMART trial**

| Measure | T0  baseline | T1  1 month | T2  3months | T3  6months |
| --- | --- | --- | --- | --- |
|  |  |  |  |  |
| Background information | **X** |  |  |  |
| Measures taken as part of usual care |  |  |  |  |
| Performance Status Scale (PSS) | **X** | **X** | **X** | **X** |
| Maximal incisor opening (mouth opening) | X | X | X | X |
| Functional Intra-oral Glasgow Scale | X | X | X | X |
| 100ml Water swallow test | **X** | **X** | **X** | **X** |
| Additional Measures for Trial |  |  |  |  |
| MD Anderson Dysphagia Inventory | **X** | **X** | **X** | **X** |
| General Self Efficacy Scale | **X** |  |  | **X** |
| Self Reported Adherence question |  | **X** | **X** | **X** |
| Functional Assessment of Cancer Therapy | **X** | **X** | **X** | **X** |
| Modified Barium Swallow Impairment Score and Penetration Aspiration score |  |  |  | X |
| Acceptability to participation and randomisation Questionnaire |  |  |  | **X** |
